# Transanal Irrigation for Neurogenic Bowel Disease, Low Anterior Resection Syndrome, Faecal Incontinence and Chronic Constipation: A Systematic Review

**DOI:** 10.3390/jcm10040753

**Published:** 2021-02-13

**Authors:** Mira Mekhael, Helle Ø Kristensen, Helene Mathilde Larsen, Therese Juul, Anton Emmanuel, Klaus Krogh, Peter Christensen

**Affiliations:** 1 Department of Surgery, Aarhus University Hospital, DK8200 Aarhus, Denmark; hellkren@rm.dk (H.Ø.K.); hemala@rm.dk (H.M.L.); therjuul@rm.dk (T.J.); petchris@rm.dk (P.C.); 2 Danish Cancer Society Centre for Research on Survivorship and Late Adverse Effects after Cancer in the Pelvic Organs, DK8200 Aarhus, Denmark; klaukrog@rm.dk; 3 Department of Clinical Medicine, Aarhus University, DK8200 Aarhus, Denmark; 4 GI Physiology Unit, University College London Hospital, London NW1 2BU, UK; anton.emmanuel@nhs.net; 5 Department of Hepatology and Gastroenterology, Aarhus University Hospital, DK8200 Aarhus, Denmark

**Keywords:** transanal irrigation, neurogenic bowel dysfunction, low anterior resection syndrome, faecal incontinence, chronic constipation, bowel dysfunction, quality of life

## Abstract

Transanal irrigation (TAI) has received increasing attention as a treatment option in patients with bowel dysfunction. This systematic review was conducted according to the PRISMA guidelines and evaluates the effect of TAI in neurogenic bowel dysfunction (NBD), low anterior resection syndrome (LARS), faecal incontinence (FI) and chronic constipation (CC). The primary outcome was the effect of TAI on bowel function. Secondary outcomes included details on TAI, quality of life (QoL), the discontinuation rate, adverse events, predictive factors for a successful outcome, and health economics. A systematic search for articles reporting original data on the effect of TAI on bowel function was performed, and 27 eligible studies including 1435 individuals were included. Three randomised controlled trials, one non-randomised trial, and 23 observational studies were included; 70% of the studies were assessed to be of excellent or good methodological quality. Results showed an improvement in bowel function among patients with NBD, LARS, FI, and CC with some studies showing improvement in QoL. However, discontinuation rates were high. Side effects were common, but equally prevalent among comparative treatments. No consistent predictive factors for a successful outcome were identified. Results from this review show that TAI improves bowel function and potentially QoL; however, evidence remains limited.

## 1. Introduction

Transanal irrigation (TAI) has received increasing attention as a treatment option in patients with bowel dysfunction as it has shown to improve faecal incontinence (FI) and chronic constipation (CC) [1,2]. With TAI, water is introduced into the bowel through the anus, facilitating emptying of the rectosigmoid and the left colon [3]. By performing regular irrigations, control of bowel function including time and place of bowel movements can be re-gained [4]. In patients with FI, efficient and controlled emptying of the bowel can be achieved with TAI. This can prevent episodes of incontinence in between irrigations for an average of two days. In patients with CC, regular evacuation of the rectosigmoid with TAI can prevent constipation [3].

TAI is introduced when conservative treatment fails. At present, TAI is the only minimally invasive treatment option for bowel dysfunction. This has positioned TAI as an important treatment modality before introducing more invasive methods such as sacral nerve stimulation, antegrade colonic irrigation or stoma formation [5].

Neurogenic bowel dysfunction (NBD) affects quality of life (QoL) negatively and is highly prevalent in patients with neurological disorders [1,4]. NBD is caused by neurological disorders such as spinal cord injury (SCI), multiple sclerosis (MS), spina bifida (SB) and Parkinson’s disease. FI and CC are very common symptoms in patients suffering from NBD with a prevalence between 23 and 80% depending on the underlying neurological disorder [1]. Patients with SCI report that bowel dysfunction is the most important problem among a wide variety of other sequelae [6]. TAI was introduced into the treatment algorithm of NBD after a randomised controlled trial (RCT) among adult patients with SCI found it to be superior to conservative treatment [7].

TAI has also shown to improve symptoms of low anterior resection syndrome (LARS) [8]. LARS is a defaecation disturbance experienced by up to 80% of patients following low anterior resection for rectal cancer [9]. The syndrome comprises a cluster of FI, emptying difficulties, urgency, increased stool frequency, variable and painful stools, altered stool consistency and soiling [5]. Fifty percent of patients undergoing low anterior resection are affected by severe LARS in the long term, which has a major impact on QoL [10,11].

FI and CC of other origin may also be improved by TAI [12]. This includes among others FI and CC caused by anorectal, gynaecological or urological surgery; prolapse disease; medication; diabetes mellitus or idiopathic FI or CC. Among patients with these diseases, bowel dysfunction also has a significant negative impact on QoL [13].

Even though TAI has been proposed for the managing of bowel dysfunction for decades, the treatment is still not well known or well established. Within the past ten years [12,14], no systematic review has been conducted across NBD, LARS, and FI and CC of heterogeneous origin. We believe that such a review would help disseminate current knowledge on the effect of TAI and be beneficial to patients suffering from NBD, LARS, and FI and CC of other origin.

The aim of this systematic review was to evaluate the effect of TAI in the management of bowel dysfunction in adults with NBD, LARS, and FI and CC of other origin.

## 2. Materials and Methods

This review was conducted according to the PRISMA guidelines [15], and the protocol was registered with the International Prospective Register of Systematic Reviews (PROSPERO) (CRD42020206262).

### 2.1. Inclusion and Exclusion Criteria

The review included all study designs reporting original data on the effect of TAI on bowel function for individuals with (1) neurogenic bowel disorders (SCI, cauda equina syndrome, MS, Parkinson’s disease, cerebrovascular events, cerebral palsy and SB), (2) low anterior resection syndrome, and (3) FI and CC of heterogeneous origin. The study population included adults (≥18 years), and only articles in English published in peer-reviewed journals were reviewed. Articles were excluded if patients were treated with any other interventions than TAI, if TAI patients were pooled with other treatment modalities, or if enemas were not clearly defined as an irrigation volume ≥150 mL.

### 2.2. Outcomes

The primary outcome for this review was the effect of TAI on bowel function measured by patient-reported outcome measures (PROMs), objective measures of bowel symptoms or compliance as a surrogate measure of clinical benefit on bowel function. Secondary outcomes included details on TAI, QoL, discontinuation rate, adverse events, predictive factors and health economics. Articles with other outcomes were excluded. Studies were defined as having short-term follow-up (FU) if FU was <12 months, as long-term if FU ≥ 12 months, and mixed if patients with both short-term and long-term FU were included.

### 2.3. Search Strategy and Data Extraction

**Figure 1:**
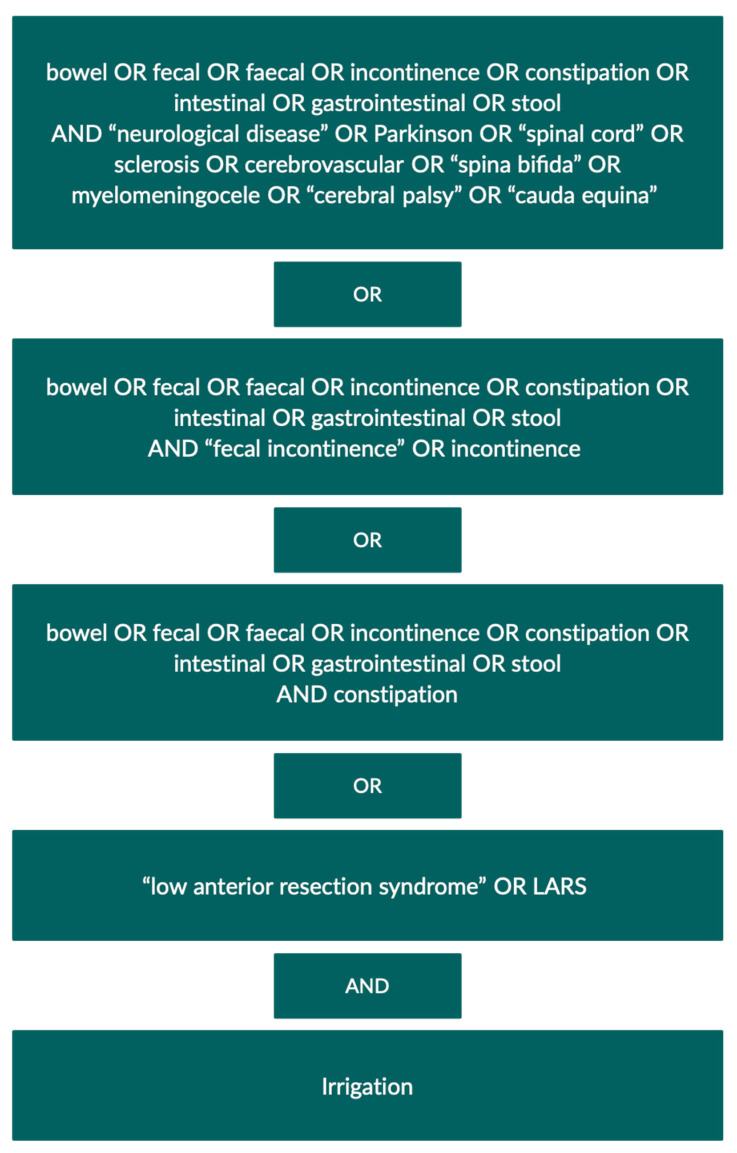
Search strategy.

On October 15, 2020, the electronic databases PubMed, Embase, and Cochrane Library were systematically searched for relevant studies. The search strategy was developed by all authors in collaboration with a librarian with expertise in systematic reviews. The search was performed using relevant MeSH- or Emtree terms and text words. The search strategy is presented in Figure 1. Covidence was used for the removal of duplicate publications, article screening and data extraction [16], and Web of Science was used to screen references and citing articles of all included studies.

Two authors (H.Ø.K. and M.M.) independently extracted information on author, study design, study population and outcomes of interest using an electronic spreadsheet in Covidence. Any disagreements during the screening or data extraction process were solved by consensus discussions between H.Ø.K. and M.M. or by a third party (T.J., K.K. or P.C.).

### 2.4. Risk of Bias and Quality Assessment

The risk of bias was assessed using a modified version of the Downs and Black checklist [17]. The checklist is validated for both RCTs and non-randomised studies [17]. It comprises 27 items covering reporting, external and internal validity, and statistical power. In the present version, item 27 addressing statistical power was modified so that a study was given one point if a power calculation was conducted and zero if it was not. For each question, one point was awarded if the study fulfilled the question (item 5 ranges from 0–2 points). Hence, the maximum score for randomised trials was 28 and non-randomised studies 25. Studies were classified as being excellent (26–28), good (20–25), fair (15–19) or poor (≤14) [18]. The assessment was independently performed by two reviewers (H.Ø.K. and M.M.). Disagreements were solved by consensus discussion between the two authors or by a third party (T.J.).

### 2.5. Data Synthesis

Results are presented separately for NBD, LARS, and FI and CC of heterogeneous origin. If data regarding NBD or LARS were separately presented in articles reporting data on FI and CC of heterogeneous origin, results were presented along with NBD or LARS results. Study and patient characteristics, details on TAI, primary and secondary outcomes, and quality assessment of each study are presented in tables and summarised descriptively. Due to the heterogeneity of outcomes and study designs, a meta-analysis was not conducted.

## 3. Results

**Figure 2:**
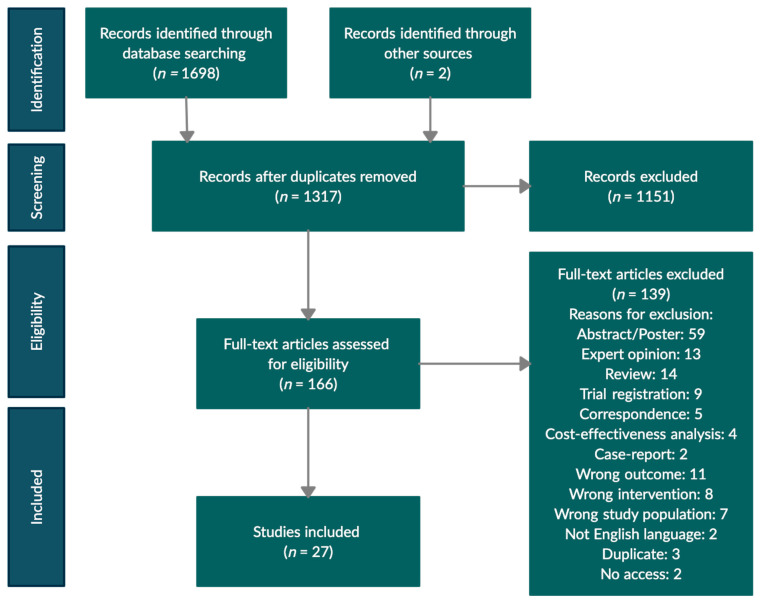
Flow diagram adapted from PRISMA [15].

In total, 1698 studies were identified through the database search. Another two studies were identified through the screening of references from the included studies. After the removal of 383 duplicates, the remaining 1317 studies were screened by title and abstract independently by two authors (H.M.L. and M.M.). As a result, 1151 studies were excluded, leaving 166 studies for full-text screening. Full-text screening was completed independently by two authors (H.M.L. and M.M.). Twenty-seven studies met the inclusion criteria. A flowchart of the screening process is presented in Figure 2.

### 3.1. Neurogenic Bowel Dysfunction

In total, eleven studies were identified reporting data on the effect of TAI in NBD patients [7,19,20,21,22,23,24,25,26,27,28]. The results are presented in Table 1. The articles were published between 2004 and 2019, and included one RCT [7], eight prospective cohort studies [19,20,21,22,23,26,27,28], one cross-sectional study [25] and one retrospective study [24]. Six studies included patients with various neurological disorders, primarily SCI [7,19,20,21,22,24]; two studies included patients with SCI [23,25]; two studies included patients with MS [26,27]; and one study included patients with SB [28]. Eight studies only included patients using TAI [19,20,21,22,23,24,26,27], one study randomised to TAI or conservative treatment [7], and two studies included patients using conservative treatment, TAI or had surgical treatment [25,28]. In total, 308 patients using TAI were included with between 4 and 62 patients included in each study. Six studies had short-term FU ([7,19,20,21,23,26], one had long-term FU (≥12 months) [27], two had mixed FU [22,24] and two studies did not report FU [25,28].

One study was assessed to be of excellent methodological quality [7], six of good quality [20,21,23,26,27,28], two of fair quality [24,25] and two of poor quality [19,22].

The predominant symptoms were FI (13–33%) and CC (55–84%) [7,20,21,23,24,27]. Irrigation volume ranged between 200 mL and 1500 mL [7,20,21,23,24,26]. Irrigation every second day was most common, and 21 to 100% of patients self-administered TAI [7,20,21,23,24,26,27]. One study reported the mean (standard deviation, SD) daily time spent on bowel management to be 47.0 (25.0) min [7]. Another study reported a mean irrigation time of 20.3 min and a mean defaecation time of 18.3 min with 60% of patients using <30 min [24]. Eight studies reported that patients received TAI training [7,20,21,22,23,24,26,27].

Bowel function was assessed by validated PROMs in eight studies [7,20,22,23,24,25,26,27,28] and by non-validated PROMs in three [21,23]. One study did not report outcome measure [19]. Six studies used the Neurogenic Bowel Dysfunction (NBD) score [7,20,22,24,25,27,31] [34], four the Cleveland Clinic Constipation Score (CCCS) [7,20,22,26,29], three the Cleveland Clinic Incontinence Score (CCIS) [24,26,28,34,35] and three the St. Mark’s Faecal Incontinence Grading System (FIGS) score [7,20,22,30].

Eight studies measuring pre- and posttreatment scores including patients with SCI, MS or SB showed a significant improvement in bowel function [7,20,21,22,23,26,27,28]. One cross-sectional study reported a prevalence of severe NBD among TAI users of 41% and a proportion of 17% as being dissatisfied or very dissatisfied with TAI [25]. A retrospective study found a mean NBD score of 6.25 and a mean CCIS of 0.50 among current TAI users [24]. One study showed a successful outcome in all patients [19].

Five studies reported QoL data. Three studies used validated PROMs [7,26,27] and two studies non-validated PROMs [21,23]. Two studies measuring pre- and posttreatment scores including patients with MS measured generic QoL [26,27]. One study showed no significant difference in the Short Form (36) Health Survey (SF-36) scale scores [26,36] and the other no difference in the European Quality of Life–5 Dimension (EQ-5D) score [37], but a significant improvement in the European Quality of Life Visual Analogue Scale (EQ-VAS) score [27]. One study including patients with SCI measured disease-specific QoL using the American Society of Colon and Rectal Surgeons Faecal Incontinence Score (FIQLS) [7,32]. The study showed a significant difference in the coping/ehavior and embarrassment scales, but not in the lifestyle or depression/self-perception scales between patients treated with TAI and conservative treatment [7].

The discontinuation rate ranged between 3 and 66% [7,20,21,23,24,26,27]. Reported reasons for discontinuation were expulsions of the catheter, bursting of rectal balloons, time consumption, heavy administration, dislike of treatment, adverse events and inefficacy. Two studies systematically reported the frequency of side effects with a range between 29 and 36% of patients experiencing side effects [7,23], the most frequent of which were abdominal pain, sweating/hot flushes, general discomfort, headache and perianal/anorectal pain. No studies reported health-economic results; however, two studies showed a reduction in urinary tract infections requiring treatment and reduction in contacts with health care professionals [7,27].

Using a multivariable analysis, one study identified several factors associated with a positive outcome of individual bowel scores; however, no consistent factors were identified [20]. To identify predictive factors for a positive outcome, four studies compared the compliant group with the non-compliant group; one study showed a higher proportion of patients with tetraplegia and patients depending on help in the non-compliant group [23]; one showed a higher baseline CCIS, SF-36 score and maximum tolerated volume to rectal balloon distension in the compliant group; one showed that impaired anal electrosensitivity was predictive for a successful outcome [27]; and one found no significant difference between the groups [24].

### 3.2. Low Anterior Resection Syndrome

In total, seven studies were identified reporting data on the effect of TAI in patients with LARS [38,39,40,41,42,43,44]. Results are presented in Table 2. The articles were published between 1989 and 2020. Five studies investigated TAI as a treatment for LARS [38,39,40,41,42], and two studies investigated TAI as a prophylactic treatment for LARS immediately after ileostomy closure [43,44].

#### 3.2.1. Transanal Irrigation as Treatment for LARS

One RCT and four prospective cohort studies investigated TAI as a treatment for patients diagnosed with LARS [38,39,40,41,42]. Two studies hadshort FU [41,42], one had long FU [40], one had mixed FU [39] and one did not report any FU [38]. In total, 96 patients using TAI were included, with between 10 and 33 patients in each study. Four studies reported reasons for LARS, and the primary reason for LARS was resection for rectal cancer (89%) [39,40,41,42]. One study reported the operation type. In this study, 78% of patients had a total mesorectal excision [41]. Three studies were assessed to be of good methodological quality [40,41,42], one to be of fair methodological quality [39] and one to be of poor methodological quality [38].

One study reported a mean (SD) irrigation volume of 1500 (600) mL [39] and two studies a median (range) of 900 (500–1500) mL and 450 (300–1000) mL, respectively [40,41]. Irrigation every day or every second day was most common, and all patients self-administered TAI [40,42]. One study reported a mean (SD) irrigation time of 43.9 (27.3) min [39]. In three studies, patients received TAI training [40,41,42,43,44].

Bowel function was assessed by validated PROMs in five studies [40,41,42,43,44] and by a non-validated PROM in one study [39]. One study used the William’s Incontinence score [39,45], one the CCIS [36,37,40], one used the LARS score [46,47,48] and the Memorial Sloan Kettering Cancer Centre Bowel Function Instrument (MSKCC BFI) [41,49], and one the LARS score, the FIGS score and the obstructed defaecation syndrome (ODS) score [29,42,50]. QoL was assessed using the SF-36 in two studies [32,40,41] and in one study using the European Organisation for Research and Treatment of Cancer (EORTC-QLQ-C30) questionnaire [42,51].

Comparing pre- and post-treatment scores, all studies showed a significant improvement of bowel function. One study showed a significant improvement of the mental component of the SF-36 and a non-significant improvement in the physical component [32,40]. Another study showed an improvement in four (mental health, social functioning, role emotional, and bodily pain) of eight SF-36 scales [41]. One study using EORTC-QLQ-C30 showed an improvement in VAS scores of the Global health status domain [42].

The discontinuation rate ranged between 0 and 23% [39,40,41]. Reported reasons for discontinuation were time consumption, dislike of treatment, cancer recurrence, proctitis and pain during TAI. Two studies reported side effects with a range between 29 and 62% experiencing side effects [39,41] including abdominal cramps, minor rectal bleeding, leakage after irrigation, nausea and pain at insertion.

One study investigated predictive factors for a decrease in LARS score, but found none [41].

#### 3.2.2. Transanal Irrigation as a Prophylactic Treatment for LARS

TAI compared to best supportive care as a prophylactic treatment for LARS immediately after ileostomy closure was investigated in an RCT with three months of FU [43]. Eighteen patients were randomised to TAI. One-year FU results were published later [44]. Patients were included if a low anterior resection for rectal cancer was performed. The studies were assessed to be of good methodological quality.

The irrigation volume during the trial was 1000 mL, and at 1-year FU the median (range) volume was 600 (200–1000) mL. During the trial, the median (range) irrigation time was 45 (30–60) min and all patients irrigated daily. At 1-year FU, irrigation was performed daily by 50% of patients. All patients self-administered TAI and were trained in TAI.

Bowel function was assessed by the number of defaecation episodes during the day and night and by the LARS score and the CCIS. QoL was assessed by the mental and physical components of the SF-36.

At 3 months of FU, the studies showed a significant difference between the groups in LARS score and CCIS, and in the number of defaecation episodes during the day and night. At 12 months of FU, a significant difference in the number of defaecation episodes during the day and night was observed, but no significant difference in the LARS score or CCIS was seen. At 3- and 12-months of FU, no significant difference in QoL measured by the SF-36 in patients using TAI compared with patients using best supportive treatment was observed.

After 3 months, 6% of patients had discontinued TAI; at the 1-year FU, 47% had discontinued. Among patients discontinuing at one year, 89% had discontinued because TAI was too time-consuming, and 11% had discontinued due to pain during irrigation.

### 3.3. Faecal Incontinence and Constipation

In total, ten studies were identified reporting data on the effect of TAI in patients suffering from FI or constipation of heterogeneous origin [52,53,54,55,56,57,58,59,60]. The results are presented in Table 3. The articles were published between 1996 and 2017, and included one non-randomised trial [59], seven prospective studies [19,52,53,55,56,57,60], one cross-sectional study [54] and one retrospective study [58]. Eight studies included patients with FI or CC of heterogeneous origin and seven of these studies included both patients with FI and CC or a combination [53,54,55,56,57,58], and one study included only patients with FI [52]. One study included patients with chronic idiopathic constipation [60], and one study included women with FI because of sphincter damage after birth trauma [59]. In total, 1012 patients using TAI were included with between 16–507 patients in each study. Two studies had short FU [19,60], three studies long FU [54,55,58] and five studies mixed FU [52,53,56,57,59].

Seven studies were assessed to be of good methodological quality [54,55,56,57,58,59,60], one of fair methodological quality [53] and two of poor methodological quality [19,52].

In four studies, irrigation volume ranged between 500 and 2200 mL [53,54,56,57] and one study reported a mean (SD) of 1750 (790) mL [55]. Irrigation every day or every second day was most common [52,53,54,55,56,60], and one study reported 99% of patients to self-administer [57]. One study reported a mean (SD) irrigation time of 36.39 (16.02) min [55] and two studies a median (range) time of 30 (10–90) min and 20 (15–30) min [52,57], respectively. In seven studies, patients received TAI training [52,53,55,56,57,58,59].

In four studies, validated bowel-specific PROMs were used as an outcome measure [55,56,57,59]; in five studies, non-validated PROMs were used [19,52,53,54,60]. One study used compliance as an outcome measure [58]. Two studies used the CCIS [57,59], one the CCCS [55], one the FIGS score [57], one the Park’s score [55], one the obstructed defaecation syndrome (ODS) score [50,57] and one the FIQL score [56]. QoL was measured in four studies. One measured generic QoL with the SF-36 [32,55], one used the disease-specific FIQLS and two used non-validated PROMs [53,57].

Three prospective studies including patients with FI and CC of heterogeneous origin showed a significant improvement in bowel function with validated PROMs [55,56,57]. One of the studies showed significant improvement in QoL using the SF-36 [55] and the other an improvement in QoL on a non-validated 11-point Likert scale [57]. The last study showed no significant improvement in the FIQLS [56].

In the studies using non-validated PROMs to measure bowel dysfunction, one study reported an overall satisfaction with TAI of 73% [54], and one study showed a successful response to TAI in 50% of patients [53]. Using compliance as a success criterion, one retrospective study showed that 43% still irrigated at the 1-year FU. The study reporting data on only patients with FI used a non-validated measure and reported a successful outcome in 38% of patients [52].

In patients with chronic idiopathic constipation, overall satisfaction was reported in 67% of patients [60]. In patients with FI following sphincter damage after birth, no difference was seen when comparing the baseline and termination score [59].

The discontinuation rate ranged between 8 and 57% [52,53,54,55,56,57,58,59,60]. Reasons for discontinuation were inefficacy, pain during TAI, time consumption, side effects, practical problems and disliking the treatment. Side effects were reported to range from 22 to 59% [54,55,57,58,60]. Reported side effects included abdominal cramps, leakage of irrigation fluid, bloating, anorectal pain, chills/shivering, nausea, dizziness and sweating.

Using a multivariate analysis, one study showed a significant association between satisfactory progress of the first training and TAI compliance [58]. A cross-sectional study showed higher satisfaction among younger adults <40 years [54]. One study found no association between incontinence score and anorectal physiology and a successful effect of TAI [53]. Another study found no correlation between baseline measures and duration of TAI treatment [60].

## 4. Discussion

Results from this review show that TAI is a beneficial treatment for both NBD, LARS, and FI and CC of heterogeneous origin with some studies reporting improvement in disease-specific and generic QoL. With few exceptions, the studies in this review have used TAI as second-line treatment when conservative treatment has failed. Therefore, results from this review mainly evaluate effects on bowel function among patients not responding to conservative treatment, i.e., patients with potentially more severe bowel dysfunction.

Overall, three studies were RCTs [7,42,43] and 16 prospective cohort studies reporting pre- and post-treatment analysis of bowel function [20,21,22,23,26,27,28,39,40,41,44,53,55,57,59,60]. One study was assessed to be of excellent methodological quality [7] and 18 to be of good methodological quality [20,21,23,26,27,28,40,41,42,43,44,54,55,56,57,58,59,60]. Except from two studies [56,59], all prospective studies comparing pre- and post-treatment scores found a significant improvement in bowel function. Two RCTs supporting the superiority of TAI compared with conservative treatment have been published [7,44]; one in patients with SCI and one as a prophylactic treatment against LARS immediately after ileostomy closure. Another RCT including patients with LARS found a significant improvement in the TAI group, but not in the tibial nerve stimulation group [42].

Change in bowel function and QoL was primarily measured with PROMs. PROMs allow for the evaluation of patients’ perspectives on functionality and QoL [62] and have gained acceptance within this research field. The use of validated instruments has previously been identified as a limitation in TAI research [12]. Overall, 67% of the included studies used at least one validated bowel-specific PROM. However, 82% of studies published within the last ten years used validated measures, showing that this limitation is no longer prominent. Nine different PROMs were used to evaluate bowel function, and this inconsistency of outcome measures compromises comparability. Numerous bowel function measures exist, which have been developed and validated differently. The NBD score and the LARS score have been developed and validated to evaluate bowel function based on a correlation with QoL, whereas the CCCS and FIGS are correlated to physiological or clinical assessment. Consensus regarding core outcome measures would ensure comparability in future research.

Half of the studies measured QoL by generic and/or disease-specific QoL measures. Three studies used a disease-specific QoL measure [7,40,56] and two of these showed improvement [7,40]. Although the NBD and LARS scores are not QoL measures, their items correlate with an impact on QoL. The reported improvement of these scores in many of the included studies could therefore suggest an improvement in disease-specific QoL. Some studies showed improvement in generic QoL measured with SF36, EQ-5D, or EORTC-QLQ-C30 [27,40,41,42,55], while other studies showed no significant change [26,43,44,56]. Two of the studies showing no improvement in generic QoL used TAI as a prophylactic rather than a symptomatic treatment [43,44]. Four studies used non-validated questions to measure QoL; three studies showed significant improvement in QoL [21,23,57]. The wording or themes explored by generic QoL instruments might be insensitive to changes in QoL resulting from an improvement in bowel function. We encourage research into generic QoL instruments sensitive to changes in bowel function that allow for a subjective valuation of the aspects of QoL that are most important to the individual patient.

Results show a high discontinuation rate at the 1-year FU of 19 to 57%, and several studies have based effect analyses solely on patients still performing irrigation at FU. Irrigation is known to be time-consuming and may involve practical difficulties. In order to overcome these challenges, patients have to experience a beneficial effect to continue the use of TAI [12]. Therefore, many studies consider the continuation of TAI as a successful outcome, and the high discontinuation rates in the studies included in this review suggest that TAI is beneficial only for a selected group of patients.

To predict a successful outcome and target the introduction of TAI to patients most likely to benefit from treatment, predictors of discontinuation have been studied. The studies included in this review reported no consistent predictive factors for a successful outcome. Using a multivariate analysis, Bildstein et al. found the progress of the first training to be a predictive factor for a successful outcome [58]. Almost all included studies in the present review reported that patients received TAI training prior to initiation, stressing that training is considered as an important part of the process. However, it is not evident which parameters the training comprises. In our clinic, all patients are taught irrigation by a specialised nurse, and the first irrigation performed by the patient or a caregiver is carried out under supervision at the clinic. In our experience, adequate training and patient support are important factors for patient compliance. Findings in this review partially support this; however, this must be further explored in future studies. Typically, clinical factors or basic demographic variables have been studied, such as age and sex, level of injury in SCI, mobility, tumour characteristics, stoma details, anorectal physiology, baseline bowel function and QoL scores. However, a successful outcome of TAI may also depend on personal characteristics such as the psychological profile and compliance with other treatment and hospital FU [5]. Future research should be directed towards better phenotyping TAI candidates. Among possible predicting factors for a successful outcome, socio-economic factors or personality traits should also be included.

Three of the major reasons for discontinuation identified through this review were technical problems, inefficacy and TAI being too timeconsuming. The primary technical problems reported were expulsion of the catheter, bursting of rectal balloons, and leakage around the catheter. Interestingly, technical problems were not reported as a reason for discontinuation amongst patients with LARS. Possible explanations might be the absence of a hyperreflective rectum in patients with LARS, which is seen in patients with NBD and can complicate rectal installation [63], or that data on technical problems was not reported.

Side effects were systematically reported in eight studies [7,23,39,40,55,57,58,60]. For NBD, side effects were reported to be experienced by 29 to 36% of patients, while this ranged between 29 and 62% for LARS and 22 and 59% for FI and CC of heterogeneous origin. There was no difference in the type of side effects reported among the different conditions. The most frequent side effects were abdominal cramps/pain, anorectal pain, nausea, sweating/hot flushes, minor bleeding and leakage of irrigation fluid. Christensen et al. reported no significant difference in the proportion of patients experiencing side effects during or immediately after TAI when comparing patients treated with TAI and those treated with conservative treatment [7]. This suggests that the side effects are not related to TAI, but to NBD itself. In SCI, autonomic dysreflexia during and after defaecation is even less pronounced when using TAI than with the usual digital manoeuvres to facilitate bowel emptying [64]. However, this finding has not been investigated for the LARS, FI or CC of heterogeneous origin. Only one study reported three serious adverse events, with no serious outcome [7], implying that such events are rare with the use of TAI. Bowel perforation is a potential risk related to TAI, and the risk has been reported to be 1 per 50,000 irrigations [65]. None of the included studies reported bowel perforations.

There are limitations to the included studies. So far, no RCTs have been conducted supporting the treatment of TAI compared with optimal conservative treatment in patients suffering from LARS, MS, FI or CC of other origin, and the risk of confounding as well as publication bias is known to be higher in non-randomised studies. FU varied between the studies, with the majority of studies having short FU time. Furthermore, conclusions may be limited by the fact that only a few studies have made power calculations, and the sample sizes of the included studies are generally modest, which may introduce type 2 errors. Generally, external validation was assessed to be of good quality in most studies; however, the modest sample size might indicate selection bias in the recruitment of patients. Systematic inclusion methods in prospective studies in the future could strengthen the evidence.

Another limitation is that many of the studies only included patients in their analysis who were still irrigating at FU. Therefore, the results primarily reflect improvements in a selected cohort. Future studies should include both intention-to-treat and per-protocol analysis. This is not necessarily a limitation; however, it should be taking into consideration when introducing TAI to patients. Since no consistent predictors supporting which patients could benefit from TAI have been identified until now, this selection process is difficult for the clinician. Therefore, a trial-and-error strategy for the introduction of TAI with focus on an individualised course of treatment has been suggested [5]. TAI is often combined with conservative modalities to optimize treatment; however, the majority of studies do not report concomitant treatment. Reporting of concomitant conservative modalities could help clinicians to optimize treatment. Another limitation to the studies is the missing reporting of clinical significance, and future studies should report results in a manner allowing for this to be assessed.

Limitations to this systematic review include a potential risk of publication bias if studies investigating TAI that found no significant results were not published. Inclusion criteria were restricted to the English language, which could have excluded relevant articles. In some early studies, different terms have been used for TAI — for example, wash-out—which were not included in the search. This may be a limitation to our search. However, we consider our search using irrigation sufficient as recent literature has used the terms TAI and rectal irrigation, which would have been included in our search. Furthermore, the literature search was limited to three databases, and additional eligible studies might have been identified through other databases.

## 5. Conclusions

Results from this review show that TAI improves bowel function and potentially improves QoL among patients with NBD, LARS, and FI and CC of heterogeneous origin; however, the evidence remains limited. Until now, the highest evidence of TAI improving bowel function and QoL is from three RCTs showing superiority of TAI over best supportive care [7,43] and TAI as more efficient than tibial nerve stimulation [42] In NBD, the majority of the evidence is for patients with SCI, MS or SB. A high discontinuation rate calls for improved patient selection to TAI. However, no consistent predictive factors for a successful outcome have been identified. In order to identify patients benefiting from TAI, a trial-and-error approach may be used to assess if patients benefit from treatment. To optimize the possibility of a successful outcome of TAI treatment, it is important to conduct a personalised treatment course with supervision from specialised health-care personnel and to monitor outcomes of TAI.

## Figures and Tables

**Table 1:** Neurogenic bowel dysfunction.

Reference	Study Design	TAI Cohort (Total Cohort)	Follow-Up Time	Inclusion Criteria	Patient Characteristics	Details on TAI	Bowel Function Outcome	Quality of Life Outcome	Discontinuation	Adverse Events	Quality Assessment ^θ^ [17]
Gardiner 2004 [19]	Prospective cohort	4	6 weeks	N/A	2 with MS, 1 with epilepsy, 1 with transverse myelitis	N/A	Successful outcome in all patients	N/A	No one discontinued	N/A	Reporting: 2 External: 1 Internal: 4 Power: 0 Total score: 7
Christensen 2006 [7]	Multicentre randomised controlled trial TAI or conservative treatment (CT)	42 (87)	10 weeks	At least 3 months after SCI Presence of one of four predefined bowel symptoms	SCI and SB Age (years), mean (SD): 47.5 (12.8) Male/female: 29/13 Predominant symptoms: CC: 76% FI: 21% Other: 3% Duration of bowel symptoms (months), median (range): 54 (4–780) American Spine Injury Association score (complete/incomplete): T9 and above: 21/10 T10-L2: 3/5 L3-S1: 1/1 S2 and below: 0/1	Peristeen^®^ (Coloplast A/S, Denmark) Volume (mL), median (range): 700 (200–1500) Frequency: 16% every day, 49% every second day, 35% 1–3 times/week 62% self-administered Trained by a specialist nurse	Termination scores: CCCS * [29], mean (SD): TAI: 10.3 (4.4) CT: 13.2 (3.4) (*p* = 0.0016) FIGS score * [30], mean (SD): TAI: 5.0 (4.6) CT: 7.3 (4.0) (*p* = 0.015) NBD score * [31], mean (SD): TAI: 10.4 (6.8) CT: 13.3 (6.4) (*p* = 0.48) Total time spent on bowel management daily (min), mean (SD): TAI: 47.0 (25.0) CT: 74.4 (59.8) (*p* = 0.040)	Termination scores, modified FIQLS * [32], mean (SD): Lifestyle: TAI: 3.0 (0.7) CT: 2.8 (0.8) (*p* = 0.13) Coping/ behaviour: TAI: 2.8 (0.8) CT: 2.4 (0.7) (*p* = 0.013) Depression/ self-perception: TAI: 3.0 (0.8) CT: 2.7 (0.8) (*p* = 0.055) Embarrassment: TAI: 3.2 (0.8) CT: 2.8 (0.9) (*p* = 0.024)	12 (29%) patients discontinued: 25% repeated expulsions of catheter, 17% prior to training, 17% lost to follow-up, 8% lack of compliance, 8% dislike of TAI, 8% burst of rectal balloons, 8% inefficacy, 8% adverse events	14 (36%) patients experienced side effects: 15.7% abdominal pain, 10.5% sweating, 7.0% chills, 5.9% pronounced general discomfort, 5.4% dizziness, 3.0% pounding headache, 2.7% flushing, 1.4% anorectal pain No significant difference in the proportion of patients experiencing side effects between the groups (*p* = 0.052) 4 adverse events in TAI group. 3 serious adverse events	Reporting: 11 External: 3 Internal: 11 Power: 1 Total score: 26
Christensen 2008 [20]	Multicentre prospective cohort	62 42 overlap- ping with Christensen 2006 [7]	10 weeks	At least 3 months after SCI Presence of one of four predefined bowel symptoms	SCI and SB Age (years), mean (range): 47.5 (25–76) Male/female: 45/17 Predominant symptoms: CC: 76% FI: 18% Other: 6% Duration of bowel symptoms (months), median (range): 60 (4–776) Complete/incomplete: 37/25 Level of injury: Supraconal: 61 Conal/cauda equina (S2–S4): 1	Peristeen^®^ (Coloplast A/S, Denmark) Volume (mL), median (range): 650 (0–1500) Frequency: 20% every day, 48% every second day, 30% 1–3 times/week, 2% never In patients irrigating daily, 40% need assistance; 60% of those who irrigated every second day needed assistance Trained by a specialist nurse	Post-treatment–pre-treatment score, mean (95% CI): CCCS: −3.4 (−4.6; −2.2) (*p* < 0.0001) FIGS score: −4.1 (−5.2; −2.9) (*p* < 0.0001) NBD score: −4.5 (−6.6; −2.4) (*p* < 0.0001)	N/A	17 (27%) patients discontinued: 29% repeated expulsions, 24% lost to follow-up, 12% prior to training, 12% inefficacy, 6% leakage of water around catheter, 6% dislike of treatment, 6% bursts of rectal balloons, 6% adverse events,	N/A	Reporting: 11 External: 3 Internal: 8 Power: 0 Total score: 22
Del Popolo 2008 [21]	Multicentre prospective cohort	33	3 weeks	Congenital SCI or acquired SCI at least 6 months previously Severe NBD with unsatisfactory bowel management	SCI, MS and SB Age (years), median (SD): 31.6 (13.3) Male/female: 18/15 Predominant symptoms: FI: 13% CC: 84% Not recorded: 3% Complete/incomplete: 13/14	Peristeen^®^ (Coloplast A/S, Denmark) Volume (mL), mean (SD): 789 (222) Frequency: 15% ≥1 time a day, 55% every second day, 30% 1–3 times a week 100% self- administered Trained by a specialist nurse	Pre/post-treatment: Likert like scale: Abdominal discomfort (*p* < 0.001) Incomplete evacuation (*p* < 0.001) Leakage of faeces (*p* = 0.002) Gas incontinence (*p* = 0.002) 11-point Likert scale: Increase in opinion of bowel function *p* = 0.001 Defaecation time: Decrease in time spent on evacuation *p* = 0.004	11-point Likert scale: Increase in QoL score *p* = 0.001	1 (3%) patient discontinued: 3% lost to follow-up	No adverse events recorded	Reporting: 10 External: 3 Internal: 7 Power: 0 Total score: 20
Loftus 2012 [22]	Prospective cohort	11	3–28 months	NBD Unsatisfactorily treated with conservative management	SCI and SB Age (years), mean (range): 44 (27–72) Male/female: 7/4 Complete/incomplete: 4/5 Level of injury: 1 C4, 2 C7, 1 T4, 1 T5, 2 T6, 2 L1	Peristeen^®^ (Coloplast A/S, Denmark) Trained by a specialist nurse	Post-treatment–pre-treatment score, mean: CCCS: −7.55 (*p* < 0.001) FIGS score: −5.36 (*p* < 0.001) NBD score: −10.32 (*p* < 0.005)	N/A	N/A	No major adverse events	Reporting: 7 External: 3 Internal: 4 Power: 0 Total score: 14
Kim 2013 [23]	Multicentre prospective cohort	52	6 months	SCI at least 6 months previously Unsatisfactorily treated with conservative management	SCI Age (years), median (range): 45.5 (18–65) Male/female: 41/10 Predominant symptoms, multiple choice: FI: 29% CC: 54% Pain/discomfort during defaecation:38% Haemorrhoid or anal bleeding: 35% Autonomic dysreflexia: 17% Injury type: Tetraplegia: 28 Paraplegia: 24	Peristeen^®^ (Coloplast A/S, Denmark) Volume (mL), mean (SD): 789 (153) Frequency: 11% every day, 17% every second day, 72% twice every week 33% self-administered Trained by an investigator	Pre/post-treatment: Self-reported impact of bowel function on QoL increased measured with a ICF qualifier scale * [33] (*p* = 0.003) Decreased defaecation time (*p* = 0.003) At 6 months FU: Satisfaction of TAI (10-point Likert scale (10 = perfect satisfaction), mean (SD): 8.33 (1.37)	At 6 months FU: Impact of TAI on QoL (10-point Likert scale (10 = perfect satisfaction), mean (SD): 8.44 (1.34)	34 (66%) patients discontinued (reasons, multiple choice): 26% time-consuming, 25% personal reasons, 24% inefficacy, 15% adverse events, 12% expulsion of catheter, 6% difficulties cleaning up after TAI, 6% dislike of treatment, 3% leakage of irrigation fluid	15 (29%) patients experienced side effects: 17% abdominal pain or discomfort, 6% minor anal bleeding, 2% hot flash, 2% headache, 2% perianal discomfort, 2% perspiration, 2% general discomfort, 2% fatigue	Reporting: 11 External: 3 Internal: 9 Power: 0 Total score: 22
Hamonet-Torny 2013 [24]	Retrospective	16	Mean (range): 31 (7.5–66) months	Patients benefitting from TAI	SCI, MS, SB, multiple system atrophy Age (years), mean: 49 Predominant symptoms: CC: 75% CC + FI: 19% CC + perianal pain: 6% Injury type: Tetraplegia: 3 Paraplegia: 2	Peristeen^®^ (Coloplast A/S, Denmark) Volume (mL), mean: 922 Mean irrigation frequency: twice a week 38% self-administered Irrigation time (min), mean: 20.3 Time to obtain defaecation after irrigation (min), mean: 18.33 Formal education, except one	NBD score, mean: 6.25 CCIS * [34,35]: 0.50 62.5% irrigated after a mean of 31 months Time spent on bowel management < 30 min for 60% of patients Difference in consumption of laxatives, mean: Before: 1.66 After: 1.4 (*p* = 0.6783)	N/A	6 (38%) patients discontinued: 50% inefficacy, 13% heavy administration, 13% vomiting following administration	1 (6%) patients experienced anal bleeding 1 adverse event	Reporting: 9 External: 1 Internal: 6 Power: 0 Total score: 16
Adriaansen 2015 [25]	Multicentre cross-sectional	29 (258)	N/A	SCI with time since injury of ≥10 years Age at injury 18–35 years Current age 28–65 years Using a wheelchair ≥ 500 m	SCI Age (years), mean (range): 45 (29–64) Male: 77% Time since injury (years), mean (range): 22 (10–46) Injury type: Tetraplegia: 12 Paraplegia: 17	N/A	Severe NBD: 41.4% Dissatisfied/very dissatisfied with TAI, 5-point Likert scale: 17.2% Perianal problems: 41.4% CC: 27.6% FI at least once a month: 34.5% Average > 60 min required for defaecation: 24.1%	N/A	N/A	N/A	Reporting: 7 External: 2 Internal: 6 Power: 0 Total score: 15
Preziosi 2012 [26]	Prospective cohort	37	6 weeks	Failure of biofeedback Not eligible for biofeedback No response to conservative treatment MS and NBD	MS Age (years), median (range): 49 (42–56) Male/female: 3/27	Peristeen^®^ (Coloplast A/S, Denmark) Recommended volume between 500–1500 mL Recommended irrigation frequency every third day adjusted according to response 93% self-administered Trained by a specialist nurse	Pre/post-treatment: CCCS, median (IQR): Pre: 12 (8.75–16) Post: 8 (4–12.5) (*p* = 0.001) CCIS, median (IQR): Pre: 12 (4.75–16) Post: 4 (2–8) (*p* < 0.001)	Pre/post-treatment: SF-36 * [36], mean (SD): Pre: 51.3 (7.8) Post: 50.4 (7.8) (*p* = 0.051)	7 (19%) patients discontinued prior to irrigation training 14 (47%) patients discontinued during trial At 6 months of follow-up, all responders continued using the irrigation, with the exception of 2 patients	N/A	Reporting: 10 External: 3 Internal: 9 Power: 0 Total score: 22
Passananti 2016 [27]	Multicentre prospective cohort	49	Minimum 1 year with a mean of 40 months	MS and NBD for ≥6 months Bowel symptoms for ≥6 months not responding to conservative management	MS Age (years), mean (range): 51 (26–80) Male/female: 12/37 Predominant symptoms: FI: 33% CC: 67%	Peristeen^®^ (Coloplast A/S, Denmark) Frequency: 48% irrigating daily, 48% every second day, 4% every third day 98% self-administered Trained by a specialist nurse	Pre/post-treatment: FI (weekly episodes), mean (range): Pre: 4.8 (1–21) Post: 0.9 (0–7) (*p* < 0.005) Severe NBD: Pre: 47% Post: 18%	Pre/post-treatment: EQ-5D * [37] utility score, mean (95% CI): Pre: 0.57 (0.5;0.65) Post: 0.52 (0.4;0.63) EQ-VAS score, mean (95% CI): Pre: 44.5 (41.26;47.73) Post: 63.4 (58.41;68.49)	22 (45%) patients discontinued: 55% dislike of treatment, 14% inefficacy, 9% adverse events, 9% other pathology, 9% lost to follow-up, 5% burst of rectal balloons	N/A	Reporting: 10 External: 3 Internal: 8 Power: 0 Total score: 21
Brochard 2019 [28]	Prospective cohort	15 (57)	Not specified for TAI group. FU for entire cohort: 46 (±36) months	Spinal dysraphism Evaluation by gastroenterologist	SB Not specified for the TAI cohort	N/A	Pre/post-treatment: Improvement of CCIS * ≥ 50%: 46.7% Variation of CCIS <50%: 19.5% (*p* = 0.016)	N/A	N/A	N/A	Reporting: 10 External: 3 Internal: 8 Power: 0 Total score: 21

^θ^ Quality assessment using a modified version of the Downs and Black checklist was performed by authors of this review. * CCCS = Cleveland Clinic Constipation score (Wexner Constipation score), FIGS = St. Mark’s Faecal Incontinence Grading System (Vaizey score), NBD = Neurogenic Bowel Dysfunction score, FIQLS = American Society of Colon and Rectal Surgeons Faecal Incontinence Score, ICF = International Classification of Function, Disability and Health scale, CCIS = Cleveland Clinic Incontinence score (Wexner Incontinence score), SF-36 = Short Form (36) Health Survey, EQ-5D = European Quality of Life—5 Dimension, EQ-VAS = European Quality of Life Visual Analogue Scale.

**Table 2:** Low anterior resection syndrome.

Reference	Study Sesign	TAI Cohort (Total Cohort)	Follow-Up Time	Inclusion Criteria	Patient Characteristics	Details on TAI	Bowel Cunction Outcome	Quality of Life Outcome	Discontinuation	Adverse Events	Quality Assessment
Iwama 1989 [38]	Prospective cohort	10	N/A	N/A	LARS 2 Turnbull-Cutait, 2 extra anal staple sutures, 1 pull-through operation, 5 anterior resections Age (years), mean (range): 61.4 (38–75) Male/female: 7/3 Predominant symptom: Frequent urge to defecate	Colostomy wash-out set (Hollister Incorporated, USA or Eisai Company, Japan) Irrigation volume (mL), range: 200–1000 Irrigation time (min), range: 20–50 Frequency of irrigation: 10% twice a day, 60% every day, 10% every second day, 20% once a week	In all cases, the frequent urge to defecate disappeared	N/A	Two patients continued using irrigation for more than 5 years, approximately once a week without any complications.	N/A	Reporting: 6 External: 1 Internal: 3 Power: 0 Total score: 10
Koch 2009 [39]	Prospective cohort	26	Mean (SD): 1.6 (1.1) years	FI after LAR for rectal cancer	LARS 30 rectal cancer Age (years), mean (SD): 67.6 (7.4) Male/female: 21/5 FU (years) after LAR, mean (SD): 4.7 (3.5)	Biotrol^®^ Irrimatic pump (B. Braun Medical A/S, Germany) Irrigation volume (mL), mean (SD): 1500 (600) Irrigation time + defaecation time (min), mean (SD): 43.9 (27.3) Frequency (day), mean (SD): 1.8 (0.7)	Pre-/post-treatment: William’s Incontinence Score * [45], mean (SD): Pre: 4.5 (0.6) Post: 1.7 (0.9) (*p* < 0.0001) 57% pseudo continent, 14% incontinent for flatus, 29% incontinent for liquid stools	N/A	5 (19%) discontinued: 10% improved and stopped TAI, 80% were not satisfied	16 (62%) patients experienced side effects: 27% abdominal cramps, 23% leakage after irrigation, 7% time-consuming, 30% other (nausea, pain inserting cone etc.)	Reporting: 10 External: 1 Internal: 8 Power: 0 Total score: 19
Rosen 2011 [40]	Multicentre Prospective cohort	14	Median (range): 29 (15–46) months	LARS Minimum 9 months after stoma reversal Insufficient conservative treatment	LARS 12 rectal cancer, 2 large villous adenomas Age (years), median (range): 68 (45–80) Male/Female: 11/3 Time (months) from LAR or stoma reversal to assessment, median (range): 19 (9–48) Neoadjuvant radiotherapy (*n*): 10	Peristeen^®^ (Coloplast A/S, Denmark) (2 used a Foley catheter) Volume (mL), median (range): 900 (500–1500) Irrigation frequency: 64% every day, 28% every second day, 7% every third day 100% self-administered Trained by a specialist nurse	Pre-/post-treatment: Defaecation episodes (n)/day, median (range): 8 (4–12) to 1 (1–2) (*p* < 0.001) Defaecation episodes (n)/night, median (range): 3 (2–5) to 0 (0–0) (*p* < 0.0001) CCIS, median (range): 17 (15–20) to 5 (4–9) (*p* < 0.01)	Pre-/post-treatment: MCS SF-36 *: 46 (35–55) to 55 (45–60) (*p* < 0.01) PCS SF-36 *: 55 (41–60) to 56 (49–62) (*p* = 0.3061) All domains of FIQLS were improved (*p* < 0.001)	No patients discontinued	3 (21%) patients experienced transient abdominal pain, 4 (29%) patients experienced minor rectal bleeding	Reporting: 11 External: 2 Internal: 7 Power: 0 Total score: 20
Martellucci 2018 [41]	Prospective cohort	33	6 months TAI following 3 months enema treatment	Short-term or long-term LARS with a LARS score ≥ 30 Failed conservative treatment	LARS 25 rectal cancer, 1 ulcerative colitis, 1 diverticular disease Age (years), median (range): 61 (29–83) Male/Female: 17/10 Neoadjuvant RT (n): 18 21 total mesorectal excision, 3 partial mesorectal excision, sigmoid resection 2, 1 total colectomy	Peristeen^®^ (Coloplast A/S, Denmark) Volume (mL), median (range): 450 (300–1000) Frequency: 3–4 times per week Trained by a specialist nurse	Pre-/post-treatment: Daily number of bowel movements, median (range): Pre: 7 (0–14) Post: 1 (0–4) Post enema: 4 (0–13) LARS score * [46,47,48], median (range): Pre: 35.1 (30–42) Post: 12.2 (0–21) (*p* < 0.0001) Post enema: 27 (5–39) (*p* < 0.0001) MSKCC BFI * [49]: Significant improvement in frequency items, urgency items, incomplete emptying, and clustering of the No difference in effect between short-term and long-term LARS	Four scales of SF-36 significantly improved (mental health, social functioning, role emotional and bodily pain).	6 (18%) patients discontinued: 17% refused participation, 50% cancer recurrence, 17% proctitis, 17% dissatisfaction with protocol 85% continued TAI after the study	N/A	Reporting: 10 External: 3 Internal: 9 Power: 0 Total score: 22
Enriquez-Navascues 2019 [42]	Randomised controlled trial TAI or percutaneous tibial nerve stimulation	13 (27)	6 months	LARS score > 29 Total mesorectal excision for rectal cancer 1 year since LARS or stoma reversal	LARS 13 rectal cancer Age (years), mean (range): 68 (48–71) Male/female: 9/4 Duration (months) of LARS, median (range): 30 (13–84) Neoadjuvant chemoradiotherapy: 6	Peristeen^®^ (Coloplast A/S, Denmark) Volume: Adjusted for each patient Frequency of irrigation: Initially once a day then adjusted to 3–4 times a week 100% self-administered Trained by a specialist nurse	Intention-to-treat: Reduction in LARS grade in at least 50% of patients: 8 out of 13 patients fell from major to minor LARS Per-protocol: LARS score, median (IQR): 35 (32–39) to 12 (12–26) (*p* = 0.021) 80% of patients treated with TAI reported a reduction of at least 50% in the FIGS score No significant improvement in the ODS * [50] score	For EORTC-QLQ-C30 * [51] VAS scores of Global health status improved (*p* = 0.020)	3 (23%) discontinued: 23% no acceptability of TAI	No significant adverse events	Reporting: 11 External: 3 Internal: 9 Power: 0 Total score: 23
Rosen 2019 [43]	Multicentre randomised controlled trial TAI or best supportive care (BS) as prophylaxisfor LARS immediately after ileostomy closure	18 (37) Rectal resection for rectal cancer	One week, 1 month, 3 months	Rectal resection for rectal cancer Anastomotic height < 5 cm above dentate line Complete healing of anastomosis Informed consent and physical and mental capability to perform TAI	LARS 18 rectal cancer Age (years), median (range): 58.5 (52–70) Male/female: 12/6 Neoadjuvant radiotherapy: 15	Peristeen^®^ (Coloplast A/S, Denmark) or Foley catheter (28 French) Irrigation volume: 1000 mL Irrigation frequency: Every 24 h Irrigation time (min), median (range): 45 (30–60) 100% self-administered Trained by a specialist	Maximum number of defaecation episodes during daytime at 1 month, median (range): TAI: 3 (1–10) vs BS: 7 (3–30) (*p* = 0.003) Maximum number of defaecation episodes during night at 3 months, median (range): TAI: 0 (0–2) vs. BS: 1 (1–5) (*p* = 0.002) LARS score at 3 months, median (range): TAI: 9 (0–34) vs. BS: 31 (3–42) (*p* = 0.001) CCIS at 3 months, median (range): TAI: 2 (0–11) vs. BS: 6 (0–17) (*p* = 0.046)	MCS SF-36 at 3 months, median (range): TAI: 55 (31–60) vs. BS: 57 (26–63) (*p* = 0.436) PCS SF-36 at 3 months, median (range): TAI: 50 (39–64) vs. BS: 51 (37–61) (*p* = 0.741)	1 (6%) patients discontinued	No complications related to TAI	Reporting: 11 External: 2 Internal: 11 Power: 1 Total score: 25
Rosen 2020 [44]	Multicentre prospective cohort	19 (37)	12 months FU from Rosen 2019 [43]	See Rosen 2019 [43]	See Rosen 2019 [43]	Peristeen^®^ (Coloplast A/S, Denmark) or Foley catheter Volume (mL), median (range): 600 (range 200–1000) Irrigation frequency: 50% every day, 30% every second day, 20% not on a regular schedule but at least 2/week. 100% self-administered	Maximum number of defaecation episodes during, median (range): Day: TAI: 3 (1–6) vs. BS: 5 (2–10) (*p* = 0.018) Night: TAI: 0 (0–1) vs. BS 1 (0–5) (*p* = 0.004) LARS score, median (range): TAI: 18 (9–32) vs. 30 (3–39) (*p* = 0.063) CCIS: TAI: 4 (0–12) vs. BS: 7 (0–16) (*p* = 0.151)	MCS SF-36, median (range): TAI: 52 (34–59) vs. BS: 56 (28–62) *(p* = 0.325) PCS SF-36, median (range): TAI: 55 (50–67) vs. 5 (31–59) (*p* = 0.460)	9 (47%) patients discontinued: 89% time-consuming, 11% pain during TAI	N/A	Reporting: 10 External: 3 Internal: 9 Power: 0 Total score: 23

* MCS = Mental Component Summary, PCS = Psychical Component Summary, LARS score = Low Anterior Resection Syndrome score, MSKCC BFI = Memorial Sloan Kettering Cancer Centre Bowel Function Instrument ODS score = the obstructed defaecation syndrome score, EORTC-QLQ-C30 = European Organisation for Research and Treatment of Cancer questionnaire.

**Table 3:** Faecal incontinence and constipation.

Reference	Study Design	TAI Cohort (Total Cohort)	Follow-Up Time	Inclusion Criteria	Patient Characteristics	Details on TAI	Bowel Function Outcome	Quality of Life Outcome	Discontinuation	Adverse Events	Quality Assessment
Briel 1996 [52]	Prospective cohort	16	Median of 18 months	Impaired continence	Heterogeneous aetiology Age (years), median (range): 52 (25–72) Male/female: 5/11 FI: 16	System unspecified Irrigation time (min), median (range): 30 (10–90) Irrigation frequency: 87% ≥ 1 time a day Trained by enterostomal therapist	38% reported a successful outcome	N/A	6 (38%) patients discontinued	N/A	Reporting: 4 External: 1 Internal: 4 Power: 0 Total score: 9
Crawshaw 2003 [53]	Prospective cohort	48	Median (range): 11 (4–27) months	Absence of correctable pathology or the failure of medical and surgical treatment	Heterogeneous aetiology Age (years), median (IQR): 54 (41–61) Male/female: 13/35 Symptoms: FI: 33 CC: 15	Equipment adapted from a Coloplast Stoma Irrigation set (Coloplast A/S, Denmark) Irrigation volume: 1500 mL Irrigation frequency: 5% twice a day, 38% daily, 17% on alternate days, 15% every 3–7 days, 19% as required Trained by specialist nurse	Bowel control, visual analogue scale: Successful response to TAI in 24 (50%) patients. Bowel rating among these 24 patients, VAS 100 maximum (100 = full control), median (IQR): Pre: 15 (3–24) Post: 50 (34–65)	QoL among 24 patients with successful outcome, median (IQR): 59.16 (46.55–67.43) No difference compared to the 24 patients without successful response	4 (8%) patients discontinued: 50% unacceptable, 50% relief of symptoms with rectopexy	N/A	Reporting: 8 External: 2 Internal: 8 Power: 0 Total score: 18
Gardiner 2004 [19]	Prospective cohort	57	6 weeks		Symptoms: FI: 16 CC: 41	N/A	Proportion of patients with successful outcome: FI: 75% CC: 51% Slow transit CC (*n* = 15): 57% Obstructed defaecation (*n* = 26): 42%	N/A	FI: 2 (12.5%) patients discontinued: 6.25% not severe enough symptoms to continue TAI, 6.25% still under review	N/A	Reporting: 2 External: 1 Internal: 4 Power: 0 Total score: 7
Cazemier 2007 [54]	Cross-sectional	40	Time (y) using irrigation, mean (range): 8.5 (2.5–18)	FI or CC TAI No response to medical treatment or biofeedback	Heterogeneous aetiology Includes NBD FI: 28 Age (years): 42 Male/Female: 5/23 CC: 12 Age (years): 45 Male/Female: 3/9	Iryflex^®^ (B. Braun Medical A/S, Germany) Irrigation volume: 500–1000 mL Frequency: 32% daily, 36% 3 times/week, 32% twice or less/week	25 (63%) patients still used TAI Overall satisfaction (*n* = 40): 29 (73%) Actual users (*n* = 25), satisfaction: 22 (88%)	N/A	Overall, 15 (38%) discontinued: FI: 5 (29%) CC: 7 (58%)	Side effects: 37.5% abdominal cramps	Reporting: 9 External: 3 Internal: 10 Power: 0 Total score: 22
Koch 2008 [55]	Prospective cohort	39	3, 6 and 12 months	FI or CC or both after failed conservative treatment or after (partially) unsuccessful surgical treatment for defaecation disorder	Heterogeneous aetiology Age (years), mean (SD): 58 (13.5) Male/Female: 13/26 Symptoms: FI: 18 CC: 11 FI + CC: 10	Biotrol^®^ Irrimatic pump (B. Braun Medical A/S, Germany) or irrigation bag Braun (B. Braun Medical A/S, Germany) 1-year FU: Irrigation volume (L), mean (SD): 1.75 (0.79) Irrigation time (min), mean (SD): 36.39 (16.02) Frequency (time/day), mean (SD): 1.1 (0.49) Trained by physician	3 months FU, number (%) pseudo continent: FI: 11 (61%) (*p* < 0.001) FI + CC: 6 (60%) (*p* = 0.009) Baseline compared with 1-year FU: FI: Park’s score [61]: 3.61 (0.5) to 1.6 (0.92) (*p* < 0.005) CCCS: Feeling of incomplete evacuation: 1.60 (2.47) to 2.75 (1.36) (*p* = 0.036)	Improvement in overall QoL measured with SF-36 and the FIQLS (*p* = 0.012)	9 (23%) patients discontinued: 78% unsatisfactory results, 22% appendicostomy	23 (59%) experienced side effects: 7% leakage after irrigation, 16% abdominal cramps, 22% abdominal bloating, 13% combination of the above side effects, 2% other	Reporting: 11 External: 2 Internal: 8 Power: 0 Total score: 21
Vollebregt 2016 [56]	Prospective cohort	60	Median FU: 12 months	Chronic defaecatory disorders not responding to conservative treatment	Heterogeneous aetiology Includes NBD and colorectal surgery Age (years), median (range): 49 (21–74) Male/female: 15/45 Symptoms: FI: 8 CC: 44 FI + CC: 8	Peristeen^®^ (Coloplast A/S, Denmark) or Biotrol^®^ Irrimatic pump (B. Braun Medical A/S, Germany) Irrigation volume (mL), median (range): 875 (250–2200) Frequency: 6% twice/day, 52% daily, 33% every second day, 6% when needed Trained by enterostomal therapist	First FU: FIQLS score did not differ between patients continuing or discontinuing TAI	First FU: Using SF-36 patients continuing TAI had more energy and were less fatigued compared with patients discontinuing TAI (*p* = 0.01) Patients continuing TAI had a tendency to have a higher SF-36 social functioning and a higher total SF-36 score, but this was non-significant	33 (55%) of patients had discontinued at the first FU, 37 (62%) at second FU and 38 (63%) at last FU	N/A	Reporting: 10 External: 3 Internal: 8 Power: 0 Total score: 21
Juul 2017 [57]	Prospective cohort	507	Mean (range): 1.06 (0.52–1.46) years	Intractable FI and/or CC with unsatisfactory results after conservative treatment	Heterogeneous aetiology Includes NBD and anorectal surgery Age (years), median (range): 56 (19–86) Male/female: 84/423 Symptoms: FI: 238 CC: 171 FI + CC: 98	Coloplast irrigation bag ^®^/Colotip^®^ (Coloplast A/S, Denmark) (majority), Coloplast irrigation bag^®^ (Coloplast A/S, Denmark)/Qufora cone^®^ (MBH International A/S), Aqua colon enema tip with silicone balloon ch 24^®^ (Runfold Plastics Ltd., UK) or Peristeen^®^ (Coloplast A/S, Denmark) Irrigation volume (mL), median (IQR): 1000 (750–1000) Irrigation time (min), median (IQR): 20 (15–30) Frequency: 35% daily, 16% every second day, 20% 2–3 times/week, 21% < once a week Self-administered 99%, assistance 1% Trained by specialist nurse	Patients with FI, pre-/post-treatment, mean change (95% CI): 11-point Likert, FI: 2.7 (2.2–3.2) (*p* < 0.001) CCIS: 2.2 (1.6–2.8) (*p* < 0.001) FIGS score: 2.2 (1.5–2.9) (*p* < 0.001) 65% improvement of FI, 29% stability, and 6% deterioration. Patients with CC, pre/post-treatment, mean change (95% CI): 11-point Likert, CC: 1.6 (0.9–2.4) (*p* < 0.001) CCCS: 1.9 (1.1–2.7) (*p* < 0.001) ODS score: 3.3 (2.0–4.5) (*p* < 0.001). 48% improvement of CC, 40% stability and 12% deterioration.	Patients with FI and CC, pre-/post-treatment, mean change (95% CI): 11-point Likert, QoL: 1.8 (1.4–2.2) (*p* < 0.001)	174 (34%) discontinued: 49% inefficacy, 18% dislike treatment, 16% symptoms resolved, 13% time consumption, 12% side effects, 8% practical problems, 21% other, 8% undetermined	120 (58%) patients experienced side effects: 23% abdominal pain, 15% anorectal pain, 6% chills/shivering, 11% nausea, 8% dizziness, 13% sweating	Reporting: 11 External: 2 Internal: 8 Power: 0 Total score: 21
Bildstein 2017 [58]	Retrospective	108	1-year FU	FI or CC Refractory to conservative treatment	Heterogeneous aetiology Includes NBD Age (years), mean (range): 55 (18–83) Male/female: 21/87 Symptoms CC: 51 FI + CC: 47 FI: 10	Peristeen^®^ (Coloplast A/S, Denmark) Trained by specialist nurse	1-year FU: 46 (42.6%) patients still irrigated 62 (57%) discontinued: 44 had discontinued, 5 failed during first training, 12 lost to follow-up and 1 died	N/A	Reasons for discontinuation: 36.4% technical problems, 40.9% inefficacy, and 22.7% constraints (primary time-consuming) Median (range) time before discontinuation: 3 (0.2–11) months	25 (54.3%) reported minor 47 minor and self-limiting adverse events: 34% leakage of fluid around catheter, 29.9% pain when inserting catheter or water, 19.1% catheter expulsion, 10.6% rectal balloon burst, 6.4% water retention	Reporting: 11 External: 3 Internal: 9 Power: 0 Total score: 23
van der Hagen 2012 [59]	Multicentre non-randomised trial	35 (70)	6 months	History of birth trauma Passive faecal incontinence CCIS ≤ 8 after anal sphincter exercise and biofeedback Defect of the internal anal sphincter	Sphincter damage after birth trauma Age (years), mean (range): 53 (38–74)	REPROP^®^ Clyster Trained by specialist nurse	In 3 (9%) patients faecal incontinence resolved completely Baseline 6-month FU: CCIS, average number of days per week with incontinence for solid or liquid stools, and average number of pads used did not change significantly	N/A	3 (9%) patients discontinued	No severe adverse effects	Reporting: 11 External: 2 Internal: 7 Power: 0 Total score: 20
Etherson 2017 [60]	Prospective cohort	102	Length of therapy use, median (range): 30.15 (1–460) weeks	Fulfilled Rome II criteria Past or present TAI treatment Received TAI for chronic idiopathic constipation (CIC) Failed all medical and behavioural therapies	Chronic idiopathic constipation (CIC) Age (years), median (range): 45 (25–84) Male/female: 7/95 Duration (years) of CIC, mean (SD): 21.8 (16.9)	Peristeen^®^ (Coloplast A/S, Denmark) (majority), Qufora ^®^ (MBH International A/S) Biotrol^®^ Irrimatic pump (B. Braun Medical A/S, Germany) Frequency: on average every second day	Overall symptom improvement: Bowel frequency: 42% Clearance of rectum: 63% Abdominal pain: 48% Bloating: 49% General well-being: 65% Awareness of urge: 25% Overall satisfaction with TAI was reported by 67% as either moderately better or very much better	N/A	48 (47%) patients discontinued	22 (22%) patients experienced side effects: 6% rectal bleeding, 3% painful irrigations, 2% painful haemorrhoids, 2% new anal fissure, 10% bursting balloons, 3% splitting of catheter	Reporting: 10 External: 2 Internal: 8 Power: 0 Total score: 20

## Data Availability

Not applicable.
